# Foot-and-Mouth Disease Virus Lacking the Leader Protein and Containing Two Negative DIVA Markers (FMDV LL3B3D A_24_) Is Highly Attenuated in Pigs

**DOI:** 10.3390/pathogens9020129

**Published:** 2020-02-17

**Authors:** Michael Eschbaumer, Veronika Dill, Jolene C. Carlson, Jonathan Arzt, Carolina Stenfeldt, Peter W. Krug, John M. Hardham, Jacob E. Stegner, Luis L. Rodriguez, Elizabeth Rieder

**Affiliations:** 1 Institute of Diagnostic Virology, Friedrich-Loeffler-Institut, Greifswald - Insel Riems 17493, Germany; veronika.dill@fli.de (V.D.); jolene.carlson@fli.de (J.C.C.); 2 Plum Island Animal Disease Center, USDA/ARS, Orient, NY 11957, USA; jonathan.arzt@usda.gov (J.A.); carolina.stenfeldt@usda.gov (C.S.); peter.krug@nih.gov (P.W.K.); luis.rodriguez@usda.gov (L.L.R.); elizabeth.rieder@usda.gov (E.R.); 3 Department of Diagnostic Medicine/Pathobiology, Kansas State University, Manhattan, KS 66506, USA; 4 Zoetis Inc., Kalamazoo, MI 49007, USA; john.m.hardham@zoetis.com (J.M.H.); c726087@gmail.com (J.E.S.)

**Keywords:** leaderless, marker virus, attenuated, foot-and-mouth disease virus, DIVA

## Abstract

Inactivated whole-virus vaccines are widely used for the control of foot-and-mouth disease (FMD). Their production requires the growth of large quantities of virulent FMD virus in biocontainment facilities, which is expensive and carries the risk of an inadvertent release of virus. Attenuated recombinant viruses lacking the leader protease coding region have been proposed as a safer alternative for the production of inactivated FMD vaccines (Uddowla et al., 2012, *J Virol* 86:11675-85). In addition to the leader deletion, the marker vaccine virus FMDV LL3B_PVKV_3D_YR_ A_24_ encodes amino acid substitutions in the viral proteins 3B and 3D that allow the differentiation of infected from vaccinated animals and has been previously shown to be effective in cattle and pigs. In the present study, two groups of six pigs each were inoculated with live FMDV LL3B_PVKV_3D_YR_ A_24_ virus either intradermally into the heel bulb (IDHB) or by intra-oropharyngeal (IOP) deposition. The animals were observed for 3 or 5 days after inoculation, respectively. Serum, oral and nasal swabs were collected daily and a thorough postmortem examination with tissue collection was performed at the end of the experiment. None of the animals had any signs of disease or virus shedding. Virus was reisolated from only one serum sample (IDHB group, sample taken on day 1) and one piece of heel bulb skin from the inoculation site of another animal (IDHB group, necropsy on day 3), confirming that FMDV LL3B_PVKV_3D_YR_ A_24_ is highly attenuated in pigs.

## 1. Introduction

Foot-and-mouth disease virus (FMDV) causes a highly contagious disease in cloven-hoofed ungulates and camelids, both in livestock (e.g., cattle, sheep, goats, pigs and Bactrian camels) and wild animals (e.g., buffalo, deer and wild boar) [1]. Chemically inactivated and highly purified whole-virus vaccines, formulated with adjuvants, are used in attempts to control the disease in many parts of the world. Mandatory use of these vaccines, in combination with the culling of infected and in-contact animals, was instrumental in the eradication of foot-and-mouth disease (FMD) from Europe and large parts of South America [2,3,4]. The production of inactivated FMD vaccines, however, is a high-risk activity, particularly in regions where FMDV does no longer occur in the wild. The inadvertent release of virus from vaccine plants caused numerous outbreaks and was a contributing factor in the decision to discontinue routine vaccination in Europe [5,6]. As recently as 2016, it was suspected that virulent FMDV had escaped from a vaccine production facility and caused an outbreak in the Russian Federation (https://www.promedmail.org/post/4662876). 

Live-attenuated vaccines are a conceivable alternative. Already successfully used in the campaigns for the global eradication of smallpox and rinderpest, live-attenuated vaccines are safer to handle than virulent virus and induce a long-lasting immunity. Historically, however, the development of live-attenuated FMDV vaccines has proven quite difficult. Intensive passaging in rodents or tissue culture lead to antigenic variation with poor immunogenicity and reversion to virulence was also a common concern [7,8]. 

More recently, genetic engineering technology has been used to create attenuated FMDV in a targeted manner [9,10]. The RNA genome of FMDV contains one large open reading frame, coding for the capsid proteins and non-structural proteins with diverse functions [11]. The first protein to be translated is the leader proteinase L^pro^, which autocatalytically cleaves from the viral polyprotein [12]. This papain-like protease is an important virulence factor that prevents the cap-dependent protein synthesis of the host by cleavage of the eukaryotic translation initiation factor 4 G (eIF4G) [13]. Available biochemical and genetic evidence shows a key role of L^pro^ in disturbing the innate immune response of the host through the inhibition of the cellular transcription factors nuclear factor κB and interferon regulatory factors 3 and 7 as well as a block of IFN-λ1 [14,15,16]. When a large part of the L^pro^ coding region is removed, the resulting “leaderless” viruses are highly attenuated in cattle and swine [7,8]. Given these observations, a marker FMDV vaccine platform lacking the leader sequence and containing specific antigenic markers in the viral 3B and 3D proteins (FMDV LL3B_PVKV_3D_YR_ A_24_ Cruzeiro, short: FMDV LL3B3D A_24_) has been developed [9]. Based on its highly attenuated characteristics, it is not currently intended to use this virus as a modified-live vaccine, but rather as a safe platform for the production of inactivated FMDV vaccine antigen.

Earlier studies have shown LL3B3D A_24_ viruses to be attenuated in cattle and pigs with no detectable viral replication, no transmission and no clinical signs in inoculated animals. However, the previous study used only two directly inoculated pigs and it did not include the double-negative marker virus (3B_PVKV_3D_YR_) [9]. Given the fact that pigs are among the most susceptible species and can shed large amounts of FMDV during infection [17], a reversion-to-virulence experiment with FMDV LL3B3D A_24_ in this species was considered. Due to the expected highly attenuated nature of the virus, however, it was first necessary to determine the optimal time points and anatomic locations for the collection of tissues needed for further passaging before proceeding with a reversion-to-virulence experiment. The present study, therefore, intended to confirm the safety of the double-negative mutant FMDV LL3B3D A_24_ in a larger cohort of animals and identify its tissue distribution in swine inoculated by either of two methods known to reliably cause disease when used with virulent FMDV: intradermal heel bulb injection (IDHB) [18] and intra-oropharyngeal deposition (IOP) [19].

## 2. Results and Discussion

### 2.1. Attenuation of Live FMDV LL3B3D A_24_ Virus in Pigs

The inoculations were carried out with no adverse events, and all 12 animals recovered from the sedation without incident. Back titration of the virus preparations used for the animal experiment revealed that the IDHB group had been inoculated with material containing 3.1 × 10^6^ pfu/mL, and the IOP group with material containing 7.7 × 10^5^ pfu/mL of live FMDV LL3B3D A_24_. These titers were about 0.5 log_10_ higher than the intended titers of 1.0×10^6^ pfu/mL and 2.0 × 10^5^ pfu/mL, respectively.

Two days after inoculation, one animal in the IDHB group (pig 6) developed a vesicular lesion surrounding the track of the inoculation needle on its right hind foot. Material from the lesion was collected at necropsy on 3 dpi. It was negative in the virus isolation assay, and the lesion was therefore deemed unrelated to the virus challenge in the sense that it was not caused by replication of FMDV in the epithelium. A possible explanation for this observation is a local infection by an environmental pathogen (e.g., fecal bacteria) that was introduced into the skin by the insertion of the needle during IDHB inoculation. The heel bulbs of the pigs had been washed, but not disinfected before inoculation. No other lesions or clinical signs of disease were observed in any animal, and no rectal temperatures higher than 40 °C were recorded at any time (mean 39.0 °C, standard deviation 0.5 °C). Body temperatures up to 40 °C (104 °F) are not unusual for young pigs, particularly during periods of excitement; therefore, the observed temperatures are not an indication of disease. 

All nasal and tonsil swabs and sera of all pigs were collected as planned. No FMDV positives were found among the nasal and tonsil swabs, indicating that no virus was shed to the environment.

Virus isolation from the collected serum samples was positive for one pig in the IDHB group (pig 05) one day after inoculation. This positive finding indicates that some virus has been released from the inoculation site into the bloodstream, perhaps through injured capillaries at the inoculation site. However, this did not lead to a generalization of the infection, and 24 h later, no detectable virus remained in the blood, nor were there any lesions or detectable virus shedding. All other serum samples from this pig and the other 11 pigs were negative in the virus isolation assay.

### 2.2. Virus Dissemination in the Inoculated Pigs

No gross lesions were detected during necropsy and there was no indication of FMD in any animal. Virus isolation from the collected tissue samples revealed one positive result in the heel bulb skin of a pig in the IDHB group (pig 3) that was sacrificed three days after inoculation. This indicates that residual inoculated virus survived at the inoculation site for 72 h or that there was limited local replication. However, there was no indication of any spread of the virus beyond that location and no sign of local replication in the skin (i.e., no visible lesion). All other tissue samples from this pig and the other 11 pigs were negative in the virus isolation assay.

### 2.3. Serology

Serum samples from all pigs taken before inoculation and on the day of necropsy were negative in the A_24_ liquid-phase blocking ELISA (titer < 1/40; data not shown), confirming that no seroconversion had occurred.

### 2.4. RNA Detection and Sequence Analysis

The positive virus isolation results for both samples were confirmed by FMDV-specific real-time RT-PCR (Table 1).

The original material of both samples contained only very low levels of FMDV genome, at or below the limit of detection of the real-time RT-qPCR. This indicates that the amount of infectious virus in these samples was very low as well, much lower than the amounts observed during infection of pigs with virulent FMDV [20]. 

**Figure 1:**
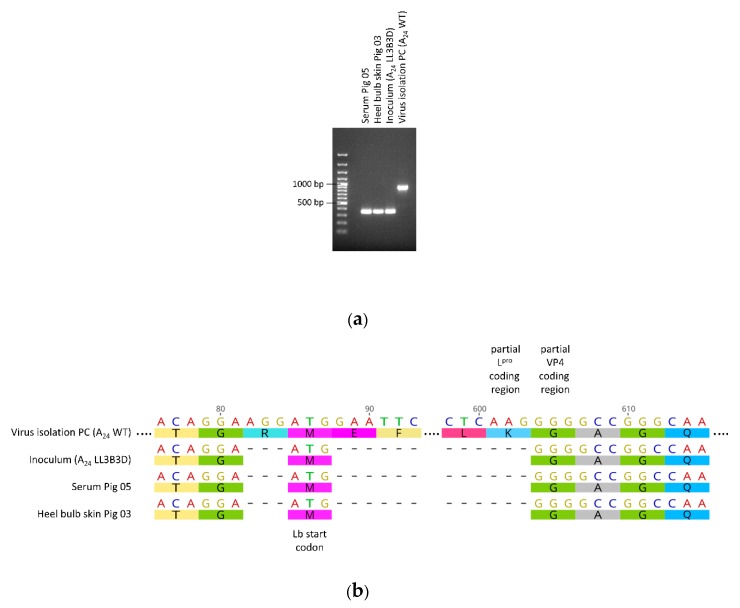
Sequencing of FMDV-positive samples. (**a**) Agarose gel image. (**b**) Excerpt from nucleotide sequence alignment. “PC”: positive control, “WT”: wild-type, “-“ deleted nucleotides.

The FMDV-positive samples from pigs 3 and 5 as well as the FMDV LL3B3D A_24_ inoculum and the parental FMDV A_24_ Cruzeiro wild-type virus were used for partial sequencing of the FMDV genome. PCR products of the expected size (867 nucleotides including the leader-coding region and 351 nucleotides without it) were obtained, their nucleotide sequences were determined and aligned (Figure 1).

The consensus sequence at the 5’ end of the open reading frame of the virus reisolated from pigs 3 and 5 was identical to the sequence of the FMDV LL3B3D A_24_ inoculum and contained the characteristic deletion of the Lb region. There was no evidence of contamination by the FMDV A_24_ WT virus used as a positive control for the virus isolation, no evidence of recombination with another virus and no evidence of any mutations in the sequenced part of the genome.

## 3. Summary and Conclusion

No clinical disease was observed in any of the pigs throughout the trial, neither after IDHB nor after IOP inoculation. No shedding of virus was observed and its dissemination in the inoculated animals was very limited; out of 264 post-challenge samples examined, only two samples (<1%) contained virus in very low amounts and only at very early timepoints after inoculation. These early re-isolations may well represent the originally inoculated virus and not virus that had undergone replication in the pigs. Overall, FMDV LL3B3D A_24_ was confirmed to be highly attenuated in pigs and it will not be necessary to carry out a reversion-to-virulence study.

## 4. Materials and Methods

### 4.1. Virus Master Seed

An FMDV LL3B3D A_24_ high-titer master stock was grown at the Plum Island Animal Disease Center (PIADC), in Orient, NY, USA in suspension baby hamster kidney cells (BHK-21) using serum-free media and a small scale bio-reactor production protocol. The master stock was verified by full-genome sequencing, clarified by centrifugation at 1000× *g* for 10 min, frozen and shipped to the Friedrich-Loeffler-Institut (FLI) in Greifswald, Germany under the necessary export license and sanitary permits.

### 4.2. Ethics Statement

All *in vivo* work occurred after ethical review and in compliance with local, state, and national animal welfare regulations. The State Office for Agriculture, Food and Fisheries of Mecklenburg-Vorpommern, the competent authority for animal experiments conducted at the Insel Riems site of the FLI, approved the experimental protocol (file no. 7221.3-1-003/17-1). The animals were handled in accordance with the applicable European and German guidelines for the use of experimental animals by researchers certified by the Federation of European Laboratory Animals Science Associations. All inoculations were performed under deep sedation and every effort was made to minimize suffering at every step of the animal experiment. 

### 4.3. Animal Trial

Twelve pigs (20–25 kg) were randomly allocated to two groups of six pigs. The groups were housed in two separate isolation rooms of the BSL4vet facility of the FLI Riems for an acclimatization period of one week before inoculation. On the day of inoculation, all animals were sedated by intramuscular injection in the side of the neck. IDHB animals were sedated with 2.6 mg of xylazine, 5.2 mg of ketamine, 1.0 mg of tiletamine and 1.0 mg of zolazepam per kg of body mass, whereas IOP animals were given 0.9 mg of xylazine, 1.9 mg of tiletamine and 1.9 mg of zolazepam per kg of body mass. Blood from the jugular vein as well as a nasal swab and a tonsil swab were taken before inoculation.

One group of pigs (n = 6) was inoculated with FMDV LL3B3D A_24_ by IOP as previously described [19]. The animals were placed in dorsal recumbency with their necks extended and 2 mL of diluted virus at a titer of 2 × 10^5^ plaque-forming units (pfu) per mL (total dose 4 × 10^5^ pfu) were deposited on the tonsil of the soft palate. Animals in the IDHB group (n = 6) received 0.4 mL of an FMDV LL3B3D A_24_ preparation with a titer of 1 × 10^6^ pfu/mL in four injections of 0.1 mL into the tarsal surface of the right hind limb [18] (total dose also 4 × 10^5^ pfu). For the inoculations, the FMDV LL3B3D A_24_ master stock was freshly thawed and diluted in sterile Minimum Essential Medium Eagle (MEM) cell culture media (cat. no. 31095029, Thermo Fisher Scientific Inc., Waltham, MA, USA) with 25 mM HEPES (cat. no. 15630049, Thermo Fisher Scientific). An aliquot of the virus preparation for each inoculation route was retained to confirm the inoculated doses with a standard plaque assay [21]. 

### 4.4. Sample Collection

After inoculation, all animals were examined daily for clinical signs of FMD and their rectal temperatures were recorded. Blood from the jugular vein as well as nasal and tonsil swabs were taken once per day. Blood was drawn into collection tubes with clotting activator. The tubes were kept at room temperature to allow the blood to clot, then the serum was separated by centrifugation (10 min, 3200× *g*, 4 °C). The nostrils were sampled with regular rayon dry swabs (Copan #155C, Hain Lifescience, Nehren, Germany), and tonsil swabs were collected with large rayon OB/GYN applicators (Puritan #808, cat. no. 10805-020, VWR, Radnor, PA, USA). Immediately after collection, nasal and tonsil swabs were soaked with 2 mL of MEM with 25 mM HEPES and 1% (v/v) antibiotic/antimycotic (cat. no. 15240062, Thermo Fisher Scientific) and transferred to the laboratory on wet ice, where tonsil swabs were centrifuged to extract the fluid from the swab tip. All samples were stored at −80 °C until further processing.

Two randomly selected animals from each group were euthanized at three days post infection (dpi) and the remaining four animals were euthanized at 5 dpi. Necropsies were performed to collect the following tissues: tongue, tonsil of the soft palate (palatine tonsil), paraepiglottic tonsil (if visible), retropharyngeal lymph node, submandibular lymph node, popliteal and inguinal lymph nodes from the right hind limb, snout skin and heel bulb skin (also from the right hind limb, corresponding to the site of inoculation). The collected tissue samples were immediately frozen at −80 °C.

### 4.5. Virus Isolation from Animal Samples

Tissue samples, swabs and serum were processed for virus isolation at FLI Riems. A piece of each tissue of approximately 50 mg was suspended in 900 µL of MEM and macerated with two 5-mm stainless steel beads for 3 min at 30 shakes/sec in a Tissue Lyser (Qiagen, Hilden, Germany). After maceration, the samples were centrifuged (2 min, 20,000× *g*, 4 °C) and 800 µL of clarified supernatant were run through a Corning Costar Spin-X filtration tube (cat. no. CLS8160-96EA, Sigma-Aldrich, Taufkirchen, Germany). 

Filtered tissue macerates, swab fluids (clarified by centrifugation at 20,000× *g* for 2 min at 4 °C) and serum samples were used to inoculate BHK-21 monolayers at 90% confluency in 25 cm^2^ tissue culture flasks. The cells used for virus isolation were adherent BHK-21 cells, clone “Tübingen”, from the Collection of Cell Lines in Veterinary Medicine at the FLI (cat. no. CCLV-RIE 164). Immediately before inoculation, the monolayers were washed with MEM with antibiotics and 1% fetal bovine serum (FBS). Two flasks per sample were inoculated with 250 µL each. The wildtype parental virus of FMD LL3B3D A_24_, FMDV A_24_/Cruzeiro/BRA/55 (short: FMDV A_24_ WT), was used as a positive control for the virus isolations.

After one hour at 37 °C, 5 mL of MEM with antibiotics and 1% FBS were added to the flasks. The flasks were kept at 37 °C for 3 days and were checked for cytopathic effect (CPE) daily. On the 3rd day, the cells were disrupted by freezing and thawing, and the lysates were passaged onto fresh BHK-21 monolayers.

### 4.6. Serology

Serum samples from all pigs taken before inoculation and on the day of necropsy were tested for FMDV-specific antibodies in a liquid-phase blocking ELISA using A_24_ WT antigen as described in the Manual of Diagnostic Tests and Vaccines for Terrestrial Animals of the World Organisation for Animal Health [22].

### 4.7. RNA Detection and Sequence Analysis

Supernatants from CPE-positive cultures and from cultures that were CPE-negative in the second passage were used for RNA extraction. Total RNA was extracted with TRIzol LS (Thermo Fisher Scientific) and the NucleoMag VET kit (Macherey-Nagel, Düren, Germany) on a KingFisher Flex magnetic particle processor (Thermo Fisher Scientific) and used for FMDV real-time RT-PCR as previously described [23]. 

RNA extracted from CPE-positive samples was additionally used for partial sequencing of the FMDV genome. Primers FMD-1174-F and FMD-2063-R [24] were used to amplify the leader-coding region of the FMDV genome with the qScript XLT One-Step RT-PCR Kit (Quantabio, Beverly, MA, USA). PCR products were screened by agarose gel electrophoresis. DNA bands were excised from the gel, purified using the QIAquick PCR Purification kit (Qiagen) according to the manufacturer’s instructions and the purified DNA was sequenced with the BigDye Terminator v1.1 Cycle Sequencing Kit (Applied Biosystems, Foster City, CA, USA). BigDye products were purified using the NucleoSEQ kit (Macherey-Nagel) according to the manufacturer’s instructions and their nucleotide sequences were determined using a 3130xl Genetic Analyzer (Applied Biosystems).

## Figures and Tables

**Table 1:** Confirmatory FMDV real-time RT-qPCR for CPE-positive samples.

Sample	Original Sample	1^st^ BHK Passage	2^nd^ BHK Passage
pig 5, serum, 1 dpi	C_q_ 36.7	C_q_ 16.6	C_q_ 17.4
pig 3, heel bulb skin, 3 dpi	no C_q_	C_q_ 29.2	C_q_ 21.2

“C_q_”: cycle of quantification, “no C_q_”: negative result.
